# Using nomograms to predict prognostic factors in young colorectal mucinous and signet-ring cell adenocarcinoma patients

**DOI:** 10.1042/BSR20181863

**Published:** 2019-07-19

**Authors:** Baochun Wang, Juntao Zeng, Yuren Liu

**Affiliations:** 1 Department of General Surgery, Hainan Province People’s Hospital, Haikou, China; 2 Department of Gastroenterology, The Third People’s Hospital of Hainan Province, Sanya, China; 3 Department of Emergency, Haikou City People’s Hospital, Haikou, China

**Keywords:** colorectal cancer, mucinous, nomogram, prognosis, signet-ring cell carcinoma, young

## Abstract

Due to insufficient quantitative evaluation of the clinic-pathological features and prognosis of young colorectal cancer (CRC) with mucinous adenocarcinoma (MAC) and signet-ring cell carcinoma (SRC), the aim of our study was to develop a nomogram to identify the prognostic predictors for overall survival (OS) in this patient population. We retrospectively evaluated the patient records of MAC and SRC patients aged ≤ 40 years. Kaplan–Meier analysis and log-rank testing were performed to estimate OS. A nomogram predicting OS was created for risk quantitation and decision tree analysis was performed for patient grouping. With a median follow-up of 36.5 months, we included a total of 90 young CRC patients for analysis. The overall cumulate 5-year OS rate was 57.7% (95% confidence interval (CI): 45.1–68.5%). The estimated 5-year OS was 62.9% (95% CI: 48.5–74.3%) for MAC and 37.3% (95% CI: 14.4–61.2%) for SRC (*P*=0.021). The recurrence rate was significantly greater in the SRC group compared with the mucinous group (52.4 compared with 26.1%, *P*=0.047). In the multivariate Cox regression model, preoperative carcinoembryonic antigen (CEA) levels and cycles of adjuvant chemotherapy (CT) were found to be an independent prognostic factor for OS (hazard ratio (HR): 2.43; 95% CI: 1.13–5.62, *P*=0.024; HR: 0.21; 95% CI: 0.083–0.57, *P*=0.002, respectively). Nomograms predicting 3- and 5-year OS were established that performed well (concordance index (c-indexes) of 0.636, 95% CI: 0.549–723) for OS. For MAC and SRC disease, a greater proportion of young patients present with advanced disease, and the prognosis for young SRC patients is poorer than MAC. Furthermore, preoperative CEA levels and cycles of adjuvant CT seem to independently affect the OS in this patient population.

## Introduction

In 2018, colorectal cancer (CRC) was the third most commonly diagnosed cancer worldwide and second in terms of mortality; in addition, it has been reported that over 1.8 million new cancer cases and 881000 deaths are estimated to occur annually, which account for 1 in 10 cancer cases and deaths [1]. Despite a slight decrease in its incidence and mortality over recent decades in China, CRC remains the fifth most common cancer, with an age standardized rate of 215.7 and 160.6 per 100000 amongst men and women, respectively [2]. In general, CRC has been regarded as a disease of older populations with more than 90% of patients diagnosed aged >55 years [3]. According to the Surveillance, Epidemiology and End Result Program (SEER) database in the U.S.A., approximately 5% of all CRC is diagnosed in patients <45 years. Furthermore, rectal cancer is diagnosed in up to 18% of patients aged <50 years [4]. In China, the age standardized rate of patients aged <30 and 30–44 years is 1.1 and 13.0 per 100000, respectively, which is significantly lower than that of patients aged 60–74 years (90.9 per 100000) [2]. However, a relative increase in the number of young patients diagnosed with CRC has been reported in many countries over the last decade [5].

Previous research has shown that young CRC patients have many unique characteristics and a relatively poorer prognosis, but these studies included all histologic types [6–10]. More recent research including 269398 CRC patients also found that young individuals with CRC have an increased risk of presenting with distant metastases [11]. However, although adenocarcinoma is the most common histologic subtype of CRC, mucinous and signet-ring cell subtypes of adenocarcinoma are seen more frequently in young patients [12]. Additionally, Tawadros et al. [13] reported that rectal cancer patients <40 years of age were 3.6-times more likely to exhibit signet cell histology. However, as far as we know, limited information is available regarding the survival and prognostic factors of young patients (≤40 years) with MAC and SRC.

A nomogram is a statistical model that combines and quantitates all proven prognostic factors using a simple graphical representation. Several nomograms for CRC prognosis have been established in recent years, but none specifically for this patient population. We thus conducted this retrospective study to quantitatively evaluate the clinic-pathological features and prognosis of young CRC patients with MAC and SRC by establishing a nomogram and decision trees.

### What does the present paper add to the existing literature?

The prognosis of MAC and SRC in young CRC patients requires elucidation. Our study shows that a greater proportion of young patients with MAC and SRC present with advanced disease. Preoperative CEA levels and cycles of adjuvant CT are two independent predictors of OS for these patients.

## Materials and methods

### Patients

The medical records of the patients who underwent surgical resection at the Third People’s Hospital of Hainan Province from 2004 to 2013 with a diagnosis of colorectal MAC and SRC were retrieved from our department. Patients who met the following criteria were included for analysis in the present study: (i) they had colorectal MAC or SRC, independently confirmed by two pathologists via H&E staining in a retrospective review; (ii) complete medical records with respect to demographic characteristics and laboratory findings, and patients had to be <40 years of age; (iii) no previous or concurrent malignancy; and (iv) follow-up for more than 6 months after the definite diagnosis. Patients were excluded if they had other types of CRC or were >40 years of age. Finally, a total of 90 patients with CRC MAC and SRC were identified and included in the present analysis. As our study was retrospective, written informed consent from patients was waved. The study was approved by the Institutional Review Board of the Third People’s Hospital of Hainan Province (2018110405).

### Clinical variables and definitions

Data were obtained by reviewing the medical records and included: gender, age at diagnosis, date of diagnosis, tumor site, pathological diagnosis, tumor stage at the time of diagnosis, adjuvant CT, cycles of adjuvant CT, preoperative CEA levels, and pathologic features (T stage, N stage, M stage, lymphovascular, and perineural invasion). All tumors were staged according to the TNM staging system of the American Joint Committee on Cancer (7th version, 2009). The tumor site was classified as colon or rectum. In particular, the cut-off point of CEA affecting survival was determined by the Cut-off Finder application. In the present study, patients ≤40 years at diagnosis were referred to as young patients, the decision of 40 years as a cutoff was based on previously published results [13–16].

### Follow-up

Patients were followed-up at 3-month intervals for 2 years, at 6-month intervals for the next 3 years, and annually thereafter. The date of the last follow-up was March 2017, which primarily consisted of telephone calls. Recurrence was determined by clinical and radiologic examination or histologic confirmation. The main pattern of recurrence was recorded as the first site of detectable failure during the follow-up period. Research end points were disease-free survival (DFS) and OS. DFS was the time from the surgery to the local or distant failure, and OS was calculated from surgery to death induced by all causes or end of follow-up.

### Statistical analysis

A comparison between means was performed using the Student’s *t* test, and proportions were compared using either the chi-square test or Fisher’s exact test, which was used for smaller numbers. Survival analysis was conducted using the Kaplan–Meier method. The comparison of the survival curves was performed using the log-rank test. A multivariable Cox regression analysis was performed to identify predictive factors of OS. Every variable was analyzed by univariate analysis, in order to cover all potentially important predictors, then variables with *P*≤0.10 in the univariate analysis were included in the multivariable analysis. This level was chosen to incorporate all potentially important predictor variables in the final modeling process. A nomogram predicting OS was constructed for young MAC and SRC patients based on the identified risk factors. Nomograms were created using the nomogram function of the ‘rms’ package in R software, and the prediction performance was assessed using Harrell’s concordance index (c-index), a main measure of discrimination. Statistical analysis was performed using SPSS 16.0 software (SPSS Inc., Chicago, IL, United States), with *P*<0.05 considered to be statistically significant. The program partykit implemented in R software (Mathsoft, Cambridge, MA) was used to generate a decision tree.

## Results

### Patients’ characteristics

A total of 90 patients met the inclusion criteria (male to female ratio, 2.1:1) and were enrolled in the present study. The baseline characteristics are listed in Table 1. The median age was 36 (range: 20–40 years), and median follow-up time was 36.5 (range: 3–109 months). A total of 65 patients received radical surgery (72.2%), and the mean preoperative CEA level was 18.5 U/ml (range: 0.2–279 U/ml). A total of 83 patients received adjuvant CT after surgery except for 7 patients, and 77 patients received more than six cycles of adjuvant CT (Table 1). The median total number of lymph nodes harvested was 17 (range: 2–33) and the number of patients with more than 12 lymph nodes was 76 (84%, Table 1).

**Table 1:** Clinicopathologic characteristics of 90 young patients with MAC and SRC

Variable	Value	Percentage
**Median age (range), y**	36 (20–40)	-
**Gender, *n***		
** Male**	69	77%
** Female**	21	23%
**Primary tumor location, *n***		
** Colon**	48	53.3%
** Rectum**	42	46.7%
**Histologic types, ***n*****		
** MAC**	69	77%
** SRC**	21	23%
**Adjuvant CT**		
** Yes**	83	92.2%
** No**	7	7.8%
**Cycles of adjuvant CT, ***n*****		
** ≥6**	76	84.4%
** <6**	14	15.6%
**Tumor size, cm**		
** ≥5**	45	50%
** <5**	45	50%
**Surgical type, ***n*****		
** Radical**	65	72.2%
** Palliative**	25	17.8%
**T stage, ***n*****		
** T1/T2**	4	4.4%
** T3/T4**	86	85.6%
**N stage, ***n*****		
** N0**	23	25.6%
** N1**	30	33.3%
** N2**	37	41.1%
**Metastasis, *n***		
** No**	69	76.7%
** Yes**	21	23.3%
**Stage, *n***		
** I/II**	15	16.7%
** III/IV**	75	83.3%
**Preoperative CEA levels**		
** Mean (range), U/ml**	18.5 (0.2–279)	-
**Lymphovascular invasion, *n***		
** Positive**	41	45.6%
** Negative**	49	54.4%
**Perineural invasion, *n***		
** Positive**	40	44.4%
** Negative**	50	55.6%

Abbreviations: n, number; y, year.

### Disease status at diagnosis

Of the 90 patients, 75 (83.3%) were diagnosed with advanced-stage disease (stages III and IV). In particular, the proportion of distant metastasis was greater in the SRC group (38.0%) compared with the mucinous group (20%). The most common site of metastasis in the SRC group was the peritoneum (*n*=4, 50%), followed by the liver (*n*=2, 25%), whereas the common site of metastasis in MAC was the liver (*n*=7, *n*=50%), followed by the peritoneum (*n*=4, 35.7%) (Table 2).

**Table 2:** Initial metastatic sites at the diagnosis

Metastatic sites	MAC, *n* (%)	SRC, *n* (%)
**Total**	14^1^	8^2^
**Peritoneum**	5 (35.7%)	4 (50%)
**Liver**	7 (50%)	1 (12.5%)
**Lung**	1 (7.1%)	0 (0)
**Bone**	1 (7.1%)	1 (12.5%)
**Lymph node**	1 (7.1%)	1 (12.5%)
**Ovary**	3 (21.4%)	1 (12.5%)

Abbreviation: *n*, number.

1 One MAC patient initially diagnosed with lymph node and liver metastasis; one MAC patient initially diagnosed with lung, liver, bone, and peritoneum metastasis.

2 One SRC patient diagnosed with liver and lymph node metastasis.

### Effect of CEA on survival

**Figure 1:**
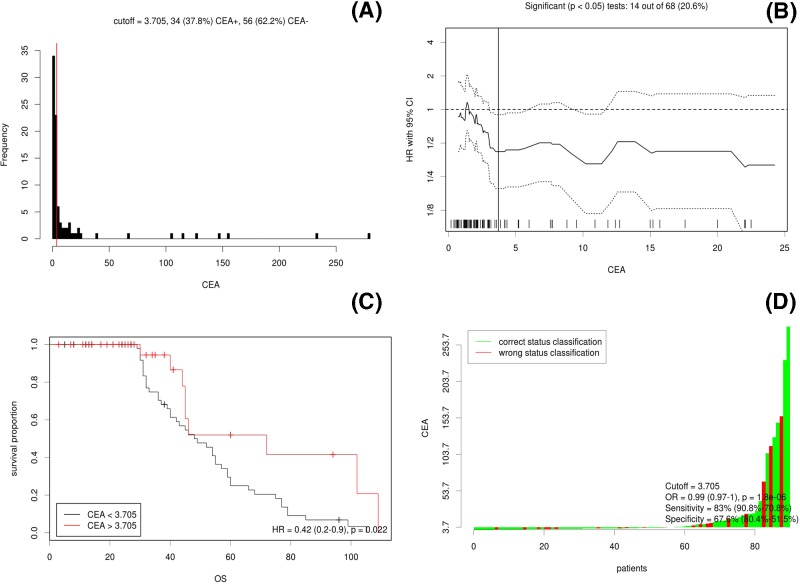
Cut-off optimization by correlation with survival detailed in the CRC data (**A**) Histograms of primary CEA levels in 90 CRC cases. Vertical line (red line) is the optimal cut-off derived from the survival-based model. (**B**) The hazard ratio including 95% CI is plotted in dependence of the cut-off. A vertical line designates the dichotomization showing the most significant correlation with OS. The distribution of CEA levels in the 90 tumors is shown as a rug plot at the bottom of the figures. (**C**) Different survival according to CEA levels; (**D**) the odds ratio including 95% CI is plotted in dependence of the cut-off. A vertical line designates the dichotomization showing the most significant correlation with OS.

The results of cut-off point determination for the primary tumor size indicated that 3.7 ng/ml was the optimal point, which was supported by multiple methods of Cut-off Finder, and the details are shown in Figure 1. The OS were significantly different between the ≥3.7 ng/ml group and the <3.7 ng/ml group (hazard ratio (HR) = 0.42, 95% confidence interval (CI): 0.2–0.9, *P*=0.0022), as seen in Figure 1.

### Pattern of failure

After a median follow-up of 36.5 months, the recurrence rate was significantly greater in the SRC group compared with the mucinous group (52.4 compared with 26.1%, *X*^2^ = 3.96, *P*=0.047). The specific sites of recurrence are listed in Table 3. The median DFS time for the SRC group was 17.0 ± 11.2 months, which was significantly shorter than the MAC group at 67.8 ± 6.0 (*P*=0.025).

**Table 3:** Pattern of recurrence

Stage	Recurrence sites	MAC, *n* (%)	SRC, *n* (%)
**I through III**	**Total**	18^1^	12
	**Local recurrence**	2 (11.1%)	5 (41.6%)
	**Liver**	4 (22.2%)	1 (8.3%)
	**Lung**	7 (38.9%)	1 (8.3%)
	**Bone**	1 (5.6%)	1 (8.3%)
	**Lymph node**	5 (27.8%)	3 (25%)
	**Ovary**	1 (5.6%)	1 (8.3%)
	**Chest wall**	1 (5.6%)	0 (0%)

Abbreviation: n, number.

1 One MAC patient relapsed with lymph node and chest wall metastasis; another MAC patient relapsed with liver, lung, and bone metastasis.

### Treatment outcome

**Figure 2:**
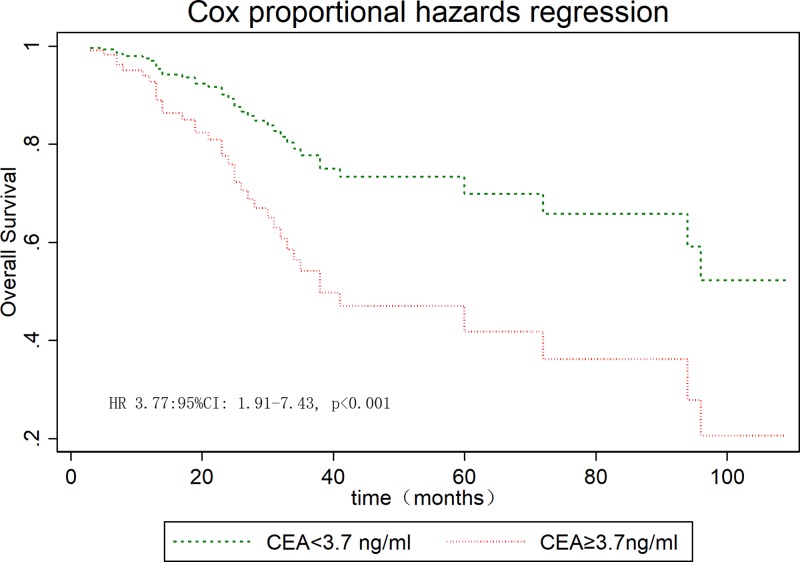
OS stratified by preoperative CEA levels in young patients

**Figure 3:**
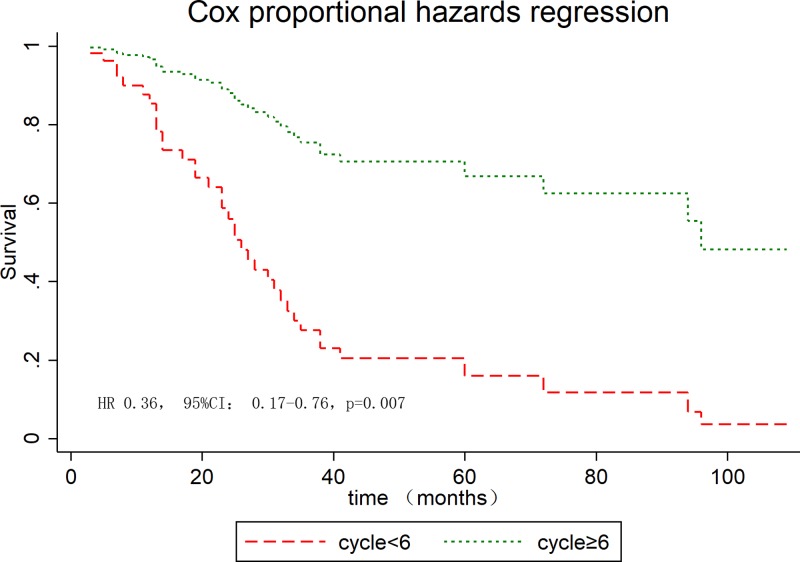
OS stratified by cycles of adjuvant CT in young patients

The median OS for the whole series was 94 months. The overall cumulate 5-year OS rate was 57.7% (95% CI: 45.1–68.5%, Supplementary Figure S1). The estimated 5-year OS was 62.9% (95% CI: 48.5–74.3%) for MAC and 37.3% (95% CI: 14.4–61.2%) for SRC (*P*=0.021, Supplementary Figure S2). The cumulative 5-year survival was 45.1% for women and 62.5% for men (*P*=0.22). The univariate analysis highlighted that metastasized disease, AJCC stage, adjuvant CT, cycles of adjuvant CT, surgery type, lymphovascular invasion, perineural invasion, preoperative CEA levels, and histologic type were significantly predictive for longer survival (Table 4), whereas gender, tumor size, and T stage were insignificant variables. In the Cox proportional hazard model, preoperative CEA levels (Figure 2) and cycles of adjuvant CT (Figure 3) were an independent prognostic factor for OS (Table 4).

**Table 4:** Predictive factors for OS using univariate and multivariate analyses of the cohort (*n*=90)

Factors	Univariate analysis	Multivariate analysis				
	HR	95% CI	*P*	HR	95% CI	*P*
**Gender**						
** Female**	1			-		
** Male**	1.58	0.82–3.04	0.17	-	-	-
**Tumor location**						
** Rectum**	1			-		
** Colon**	0.58	0.11–1.13	0.11	-	-	-
**Histologic types**						
** MAC**	1			-		
** SRC**	1.97	0.96–4.06	0.064	-	-	-
**Adjuvant CT**						
** No**	1			1		
** Yes**	0.20	0.083–0.50	0.001	0.96	0.29–3.21	0.95
**Cycles of adjuvant CT**						
** <6**	1			1		
** ≥6**	0.36	0.17–0.76	0.007	0.21	0.083–0.57	0.002
**Tumor size, cm**						
** <5**	1			-		
** ≥5**	1.42	0.91–2.23	0.13	-	-	-
**Surgical types**						
** Radical**	1			1		
** Palliative**	3.90	2.03–7.52	<0.001	1.67	0.43–6.49	0.46
**T stage**						
** T2**	1					
** T3**	1.55	0.20–12.13	0.67	-	-	-
** T4**	3.93	0.52–29.52	0.18	-	-	-
**N stage**						
** N0**	1			1		
** N1**	2.16	0.77–6.08	0.144	-	-	-
** N2**	2.89	1.07–7.76	0.035	-	-	-
**Metastasis**						
** No**	1			1		
** Yes**	4.39	2.24–8.59	<0.001	1.05	0.18–5.95	0.95
**Stage**						
** I/II**	1			1		
** III/IV**	3.25	1.83–5.80	<0.001	1.57	0.53–4.69	0.42
**Preoperative CEA levels, U/ml**						
** <3.7**	1			1		
** ≥3.7**	3.77	1.91–7.43	<0.001	2.43	1.13–5.62	0.024
**Lymphovascular invasion**						
** Negative**	1			1		
** Positive**	3.00	1.52–5.39	0.002	1.36	0.53–3.47	0.52
**Perineural invasion**						
** Negative**	1			1		
** Positive**	1.85	0.96–3.57	0.065	1.28	0.59–2.79	0.53

### Nomogram development

**Figure 4:**
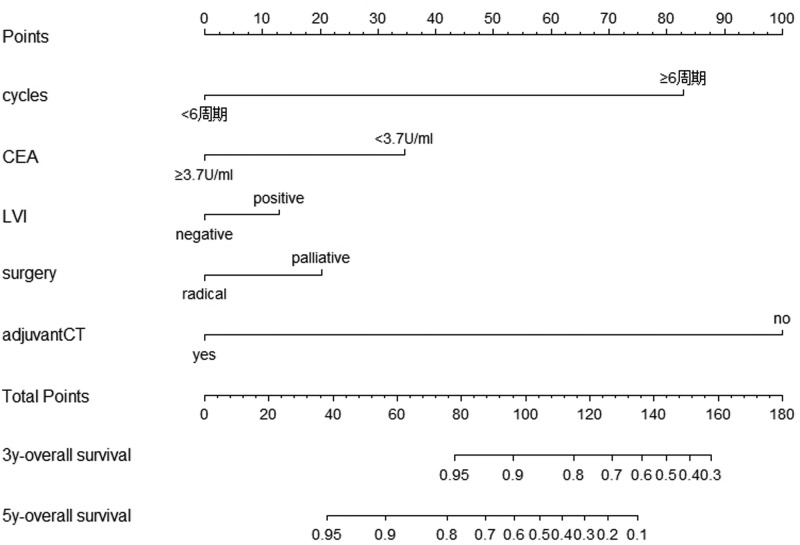
Survival nomogram of young CRC patients with MAC and SRC Nomograms for predicting the OS and each variable corresponds to a point on the scale. According to the sum of these points projected on the bottom scales, the nomogram can provide the probabilities of 3- and 5-year OS for an individual patient.

We performed univariate and multivariate analyses to screen for independent prognostic factors in order to build the nomograms. In univariate analyses, nine variables were found to be related to OS (*P*<0.05 for all; Table 4) and nomograms incorporating the respective prognostic factors of OS were established (Figure 4). To further test predictive performance, nomograms were applied to an independent validation set. The c-index of the nomograms reached 0.636 (95% CI: 0.549–723) in predicting OS.

### The classification tree for the prognosis of young MAC and SRC

**Figure 5:**
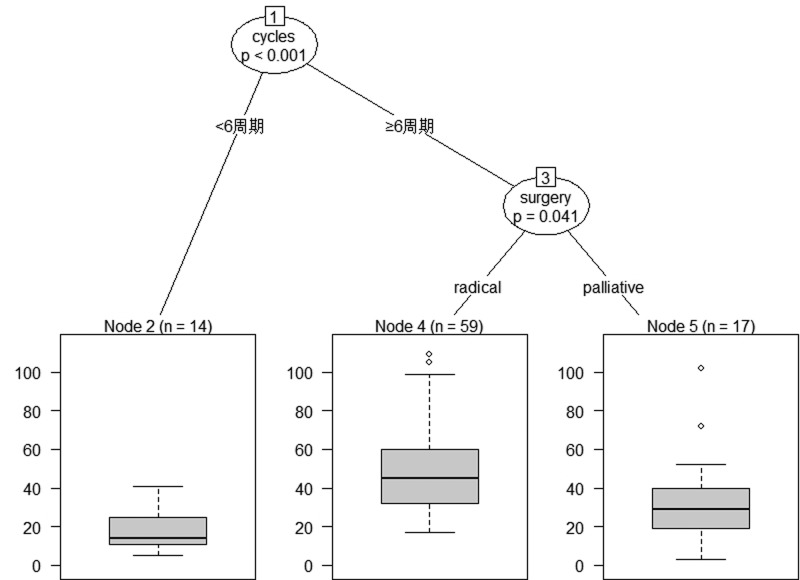
Best tree for prognosis of young MAC and SRC patients Cycles of adjuvant CT was the initial node and type of surgery followed.

Classification tree analysis was another method of obtaining the prognostic factors associated with young MAC and SRC. The result of the pruned tree is shown in Figure 5. Cycles of adjuvant CT is the initial node and the intention of initial surgery is also a determinant for prognosis of this patient population.

## Discussion

Primary colorectal MAC and SRC are two rare subtypes of CRC that are associated with a worse prognosis than classic adenocarcinoma [17–19]; however, the quantitative evaluation of clinic-pathological features and prognosis of young CRC patients with MAC and SRC is limited. Although CRC is diagnosed predominantly in older patient populations, a small number of CRC patients present at a younger age [20]. Until now, there has been no agreement regarding the cut-off age for the diagnosis of CRC in young patients. In the present study, we identified 40 years as a cut-off point, which is based on previously published results [15,16,21,22]. Our findings on gender differences are similar to published reports on CRC in <40 year olds with a male-to-female ratio of 2.1:1. On the other hand, the present study indicates that the prognosis of male patients seems to be better than for female patients, but with no significant difference (*P*=0.22). Another important finding of the present study is that young MAC and SRC patients present with advanced-stage disease. Berut et al. [3] also found that young CRC patients were often diagnosed in advanced stages. In addition, Nitsche et al. [18] also demonstrated that both MAC and SRC patients had more advanced stages of the primary tumor and lymph node involvement in comparison with adenocarcinoma. Consistent with previous results, 75 (83.3%) out of 90 patients presented with stage III and IV disease. Peritoneal dissemination is a common metastatic site of CRC, which presents in approximately 7% of CRC patients at the time of diagnosis [23]. Several risk factors for the development of peritoneal metastases have been identified, including right-sided tumor, advanced T-stage, advanced N-stage, poor differentiation grade, and younger age at diagnosis [24]. In concordance with the literature, our study finds that the peritoneum is the most common site of metastasis in young patients with SRC and MAC.

As for the outcome of patients with MAC and SRC, most published studies found that the prognosis of this patient population was very poor. Messerini et al. [25] reported the overall 5-year survival rate of those with colorectal SRC was 9.1%, and survival was significantly influenced by tumor stage. Nitsche et al. [18] found that the 5-year cause-specific survival was 61 ± 3% for MAC and 21 ± 8% for SRC. Chen et al. [19] also reported that the 5-year OS rates of patients with SRC and MAC were 11.9 and 49.4%, respectively. Lee et al. [26] reported that the 3- and 5-year survival rates in the SRC group were 33.7 and 25.3%, respectively. The OS rate of patients with SRC was significantly poorer than that of patients with mucinous or poorly differentiated adenocarcinoma. The findings of the present study are consistent with these published reports. Based on these findings, SRC patients could be regarded as a different clinical entity due to its poor prognosis [18]. Indeed, carcinogenetic factors, tumor growth, and development differ between SRC and non-SRC tumors [27]. Additionally, we conducted multivariate regression analyses and found a survival advantage for patients presenting with normal preoperative CEA levels and those who received more than six cycles of adjuvant CT. From the classification tree analysis, cycles of adjuvant CT is the initial node and the intention of initial surgery are also determinants for prognosis of this patient population. Based on our findings, sufficient cycles of adjuvant CT are recommended for young MAC and SRC patients with high-risk factors.

Distant failure has been the predominant failure pattern after the routine use of surgery and adjuvant CT in patients with locally advanced CRC. In our series, distant metastasis is the most common type of failure in MAC patients and was seen in 16 patients. The lung, liver, and lymph nodes are the most common sites of distant recurrence in patients with MAC. Interestingly, local recurrence is the most common type of failure in SRC patients and was observed in five patients. The lymph nodes are the most common sites of distant recurrence in patients with SRC and the findings of the present study are consistent with previous results. Lee et al. [26] reported that recurrence after resection in the SRC group was significantly higher than that of non-MAC (33.3 compared with 10.7%, *P*=0.038).

The current study is limited by its retrospective design, the relatively small number of young SRC and MAC patients, and possible patient selection bias. However, it appears difficult to conduct a randomized trial for this rare disease in young patients. In addition, to the best of our knowledge, this is the first study that specifically assesses the long-term survival and prognostic factors of young patients with SRC and MAC.

## Conclusion

For MAC and SRC disease, a greater proportion of young patients present with advanced disease. The prognosis of young SRC patients is poorer than MAC. The most common type of failure in young SRC is local recurrence, whereas it is lung metastasis for MAC. Furthermore, preoperative CEA levels and cycles of adjuvant CT seem to independently affect the OS in this patient population.

## Supporting information

**Supplementary Figure S1:** 

**Supplementary Figure S2:** 

## Abbreviations

CEA: carcinoembryonic antigen
CI: confidence interval
CRC: colorectal cancer
CT: chemotherapy
c-index: concordance index
DFS: disease-free survival
MAC: mucinous adenocarcinoma
OS: overall survival
SRC: signet-ring cell carcinoma
