# Supplementary figures and images for: Using nomograms to predict prognostic factors in young colorectal mucinous and signet-ring cell adenocarcinoma patients
Source: Biosci Rep. 2019 Jul 19;39(7):BSR20181863. doi: 10.1042/BSR20181863 (PMC6639454; doi:10.1042/BSR20181863)

overall survival

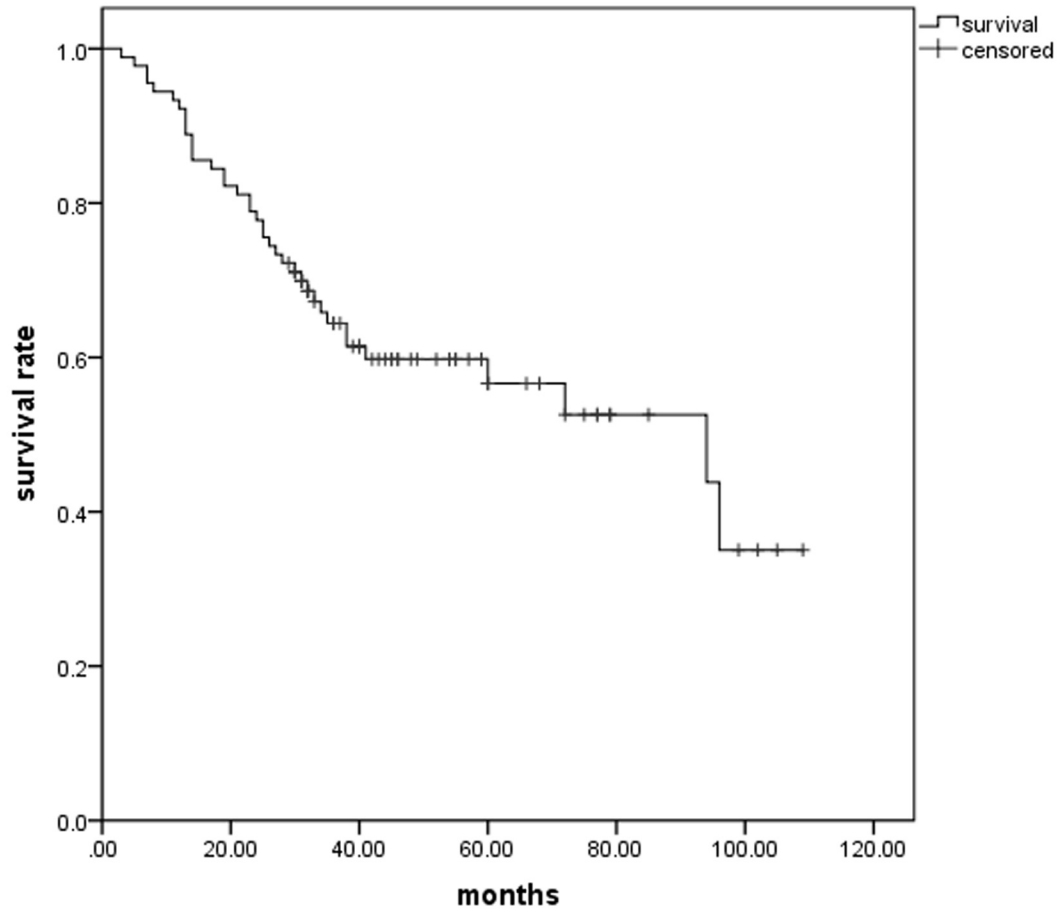

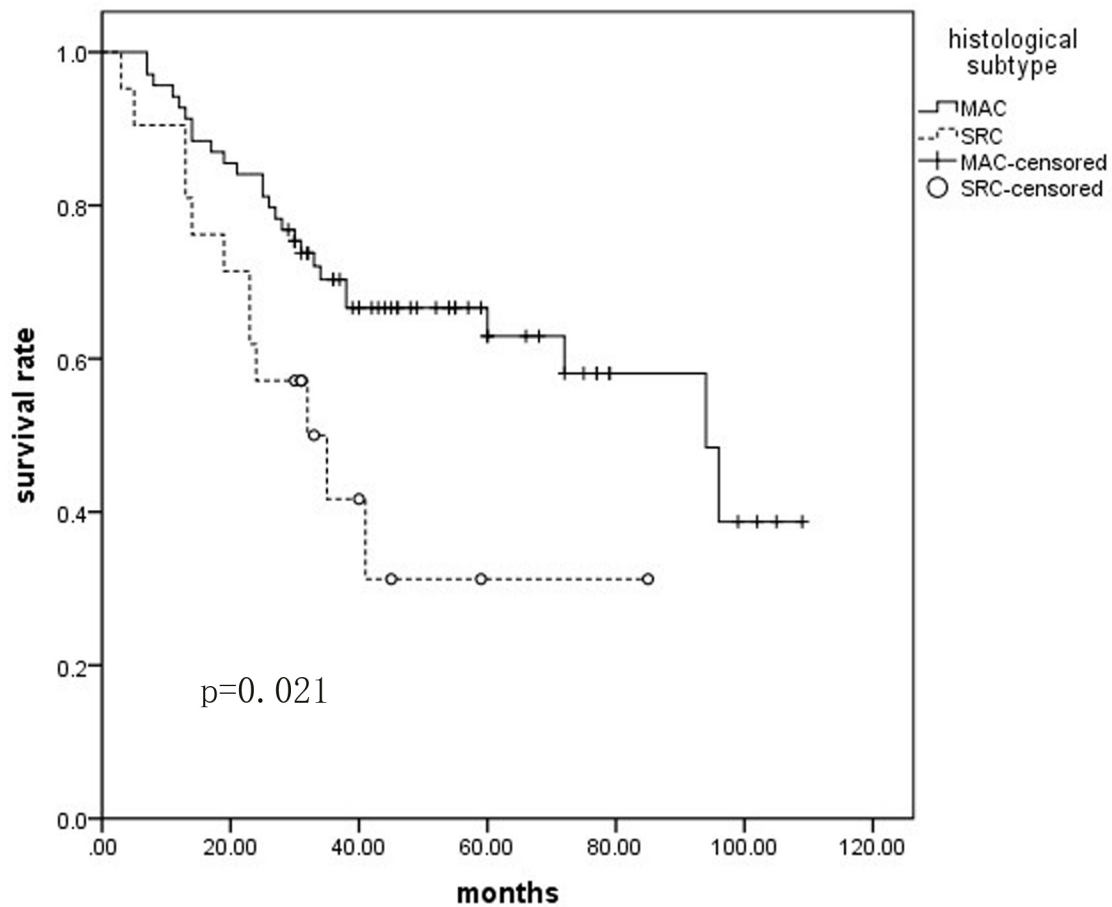

Supplement: Supplementary file 1 [file bsr20181863_Supp1.pdf]
